# Pennsylvania Hospital

**Published:** 1838-06-20

**Authors:** Henry H. Smith

**Affiliations:** Resident Surgeon


### CLINICAL REPORTS.

### PENNSYLVANIA HOSPITAL.

[*Reported by* Henry H. Smith, M. D., *resident Surgeon.*]

*List of Accidents, admitted into the Pennsylvania Hospital, from May* 30*th to June* 13*th*, 1838.

One case of contusion of the foot, caused by a fall; dressed with lead-water cloths, and fracture-box—since discharged. One case of epistaxis; the hæmorrhage had continued for thirty-six hours, previous to his entrance; arrested by plugging the nostril with lint, moistened with tinct. ferri muriat.; the patient was attacked with mania a potu, of which he died. One case of deep burn of the arms and front part of the chest, in a female aged eighty-six years, caused by her clothes taking fire; dressed with Goulard's cerate—death twelve hours after admission. One case of compound fracture of the first phalanx of the ring finger, caused by a spade falling on it; dressed with adhesive plaster, and a splint; afterwards attacked by erysipelas—poulticed, and still under treatment. One case of lacerated wound of the scalp, caused by a fall during a fit of epilepsy; dressed with adhesive strips—since discharged. One case of incised wound of the abdomen; the knife only penetrated a portion of the muscles, without involving the peritoneum; dressed with single suture and adhesive strips—nearly well. One case of fractured clavicle, caused by a fall; dressed with the usual apparatus—no deformity. One case of contusion of the shoulder and hip, caused by a fall from a tree—discharged in three days. One case of incised wound of the abdomen, caused by a stab; the external wound, of one inch and a half, was brought together by a suture, carried deeply through the muscles; death took place in seven hours after admission. On examination, the muscles, peritoneum, both sides of stomach, and pancreas, had been cut; the epigastric artery, and the vessels of the stomach, were divided, and about 42 f℥ of blood were found in the abdominal cavity. One case of contusion of the head, face, and body, received in fighting—since discharged. One case of compound fracture of the ulna, two inches above the joint, caused by a blow from a pick-axe; wound dressed with strips, and uniting; arm dressed with two splints. One case of fractured ribs, caused by a fall; dressed with a tight bandage. One case of simple fracture of both bones of the right leg, and compound fracture of both bones of the left leg, caused by falling through a hatchway; the right leg was dressed with a fracture-box, and cold applications; the wound in the left was dressed with lint, wet with white of egg, so as to form an artificial scab; limb placed in fracture-box, and kept wet with lead-water. One case of compound fracture of the skull, caused by a fall against a circular saw in a steam saw-mill; the scalp was much torn and turned back from the skull, for the distance of about six inches by four, for which distance the skull was bare; the skull was fractured, from the lower portion of the os frontis to the vertex, and, near the centre of the head, the saw had penetrated the dura mater, a small portion of which projected; no cerebral matter was seen after a careful washing of the scalp, shaving the head, and removing the pieces of bone; the wound was brought together by adhesive strips, and dry lint applied over the wound, and a bandage; it was also necessary to take up a branch of the temporal and one of the occipital arteries, to arrest the hæmorrhage, which had been considerable, previous to admission. The man died on the third day after admission, and on examination the brain was found wounded to the depth of an inch and a half, with softening around the wound for some distance. The cases reported in the last are still under treatment.

